# Chinese Medicine in the Battle Against Obesity and Metabolic Diseases

**DOI:** 10.3389/fphys.2018.00850

**Published:** 2018-07-06

**Authors:** Lingyan Xu, Wenjun Zhao, Dongmei Wang, Xinran Ma

**Affiliations:** Shanghai Key Laboratory of Regulatory Biology, Institute of Biomedical Sciences, School of Life Sciences, East China Normal University, Shanghai, China

**Keywords:** traditional Chinese medicine, artemisinin, curcumin, celastrol, capsaicin, berberine, ginsenosides

## Abstract

Obesity is a multi-factor chronic disease caused by the mixed influence of genetics, environments and an imbalance of energy intake and expenditure. Due to lifestyle changes, modern society sees a rapid increase in obesity occurrence along with an aggravated risk of metabolic syndromes in the general population, including diabetes, hepatic steatosis, cardiovascular diseases and certain types of cancer. Although obesity has become a serious worldwide public health hazard, effective and safe drugs treating obesity are still missing. Traditional Chinese medicine (TCM) has been implicated in practical use in China for thousands of years and has accumulated substantial front line experience in treating various diseases. Compared to western medicine that features defined composition and clear molecular mechanisms, TCM is consisted with complex ingredients from plants and animals and prescribed based on overall symptoms and collective experience. Because of their fundamental differences, TCM and western medicine were once considered irreconcilable. However, nowadays, sophisticated isolation technologies and deepened molecular understanding of the active ingredients of TCM are gradually bridging the gap between the two, enabling the identification of active TCM components for drug development under the western-style paradigms. Thus, studies on TCM open a new therapeutic avenue and show great potential in the combat against obesity, though challenges exist. In this review, we highlight six key candidate substances derived from TCM, including artemisinin, curcumin, celastrol, capsaicin, berberine and ginsenosides, to review their recent discoveries in the metabolic field, with special focus on their therapeutic efficacy and molecular mechanisms in treating obesity and metabolic diseases. In addition, we discuss the translational challenges and perspectives in implementing modern Chinese medicine into the western pharmaceutical industry.

## Introduction

For thousands of years, before the introduction of western medicine, Chinese people relied on traditional Chinese medicine (TCM) to treat diseases and relieve discomfort. TCM is complex formula constituted of mixed medicinal extracts from Chinese herbs and animals, and is prescribed based on the philosophical theory of Chinese medicine, for instance, the theories of “Yin Yang” and “Five Elements.” Though mysterious and empirical, TCM has gained front-line experience in combating diseases for millennia in Chinese history with effectiveness and low side effects. However, in the modern society, with the prevalence of western medicine, which is developed under strict experimental proofs and feature clear-cut molecular compositions and mechanisms of action, TCM meets serious criticisms and challenges due to undefined compositions and unclear therapeutic mechanisms (Qiu, 2007). Fortunately, nowadays, sophisticated isolation techniques have enabled scientists to identify active ingredients from TCM to elucidate the molecular mechanisms of their therapeutic effects, thus promote the re-discovery of ancient TCM compounds to be potentially developed as drugs under western medical standard. A few promising TCM compounds with successful implications include, but not limit to, artemisinin in treating malaria (Liu et al., 2006) and arsenic trioxide in acute promyelocytic leukemia (Lo-Coco et al., 2013), with more waiting to be added to the list.

Looking back into the history of human diseases, before the enlightenment of modern medicine and vaccinology, infectious disease outbreaks decimated large populations and were once the most threatening events to mankind. In today’s society, however, with large scale vaccination and a highly organized disease control system, the biggest challenge to human health has shifted to noninfectious chronic disease (NCD) like obesity, metabolic diseases, cardiovascular diseases, neurodegenerative diseases, inflammatory diseases and various types of cancer. Among them, obesity holds special importance as it is a major risk factor for many other metabolic diseases, i.e., type 2 diabetes mellitus, nonalcoholic fatty liver disease, hyperlipidemia, etc., thus renders it an effective target for disease intervention (Haslam and James, 2005). Briefly, obesity is an excessive deposit of white fat in the body. The abnormal accumulation of fat poses adverse impacts on the metabolic fitness of the body and is positively associated with aberrant metabolic complications including hyperglycemia, insulin resistance, dyslipidemia, hypertension (Bays and Dujovne, 2006) and chronic low-grade inflammation (Saltiel and Olefsky, 2017). Fat tissues are metabolically active organs and are classified as white, brown and beige fat based on their morphology, physiology and functions (Boss and Farmer, 2012; Xu et al., 2018). Together they maintain the energy balance of the body with white fat storing energy in forms of triglycerides, while brown and beige fat dissipating energy for thermogenesis *via* UCP1 activation. The disturbance of energy balance among adipose tissues may lead to increased adiposity and/or obesity.

Considering different therapeutic leads, compounds derived from TCM possess two major advantages over synthetic drugs in treating obesity. First, obesity is a complex disease that shows no clear pathogens and its pathogenesis involves multiple genetic factors and signaling networks. In patients with obesity and metabolic diseases, multiple organs, i.e., fat tissues, liver, muscle, and even brain suffer pathological changes. In this sense, the concept of TCM emphasizes the holistic treatment of a disease. Studies on TCM have demonstrated that their active ingredients target multiple organs and tissues in the body to exert systemic effects, which match the characteristics of obesity. Second, obesity and metabolic diseases are not as immediately crippling or death threatening as infectious diseases. Patients require long-term care and persistent treatment to prevent advance disease progression, which greatly burdens their family and the society as a whole. Thus, it is important to take long-term effectiveness and safety into account when developing drugs for obesity. In this regard, most TCM consists of herbal-based medicinal extracts that have been prescribed and widely used in Chinese people’s daily life for purposes ranging from health-promoting to disease treatment for thousands of years, which provide solid though empirical evidence for its safety. These characteristics make TCM a promising source for new drug development for obesity and metabolic diseases. However, putting their appealing efficacies aside, the caveat is that much of TCM has unclear compositions and vague mechanisms, which not only hinders their wide application into modern clinic, but can sometimes be detrimental. One extreme case is the recently reported implication of aristolochic acids and similar compounds from *Aristolochia* and related plant in liver cancers in Asia population for their mutagenesis attribution (Ng et al., 2017). Thus, it is vital to place TCM in the pipeline of standard western drug development: identification, isolation and/or synthesis of the active component, clarification of the molecular target and mechanism, and eventually developed as a pharmaceutical (Corson and Crews, 2007). During this process, various factors like therapeutic mechanism, clinical data, development cost, etc. all play important roles. In recent years, great interests have been focused on TCM, producing a substantial amount of research data. In this review, we highlight recent studies of six promising TCM compounds in the metabolic aspect based upon their clinical, mechanistic properties or current application, including artemisinin, curcumin, celastrol, capsaicin, berberine and ginsenosides.

## Acknowledged Vs. Controversial: Artemisinin and Curcumin

Although the interests in TCM’s potential in treating obesity and metabolic diseases only start to rise in recent years, many TCM ingredients have already been isolated and tested over the years. Substantial data demonstrates their effectiveness and low adverse effects in various diseases, promoting a few into pre-clinical or clinical trials, though not all trials gave consistent results. Among them, artemisinin and curcumin represent two different extremes, one’s efficacy is well acknowledged while the other is shrouded in controversy.

### Artemisinin

Artemisinin is derived from the sweet wormwood (*Artemisia annua* L.) that is regularly used as classic TCM. Artemisinin and its semi-synthetic derivatives-based therapy are currently the standard and the most effective treatment for uncomplicated *Plasmodium falciparum* malaria. In 2015, Dr. Youyou Tu was awarded the Nobel Prize for medicine for discovering and isolating artemisinin. This makes artemisinin the most acknowledged and successful TCM compound.

Aside from malaria, in recent years, with the spike in overweight and obesity rate, more and more attentions are focusing on the roles of artemisinin and its derivatives in treating obesity and metabolic diseases. For example, artemisinic acid and artesunate are found to inhibit the development and differentiation of adipocytes by suppressing master regulators C/EBPs and PPARγ in adipogenesis (Lee et al., 2012; Jang, 2016). In addition, since the groundbreaking discovery of brown and beige fat in human adults, activation of brown fat and browning of white fat, two major sources of adaptive thermogenesis and important outputs for energy expenditure, have emerged as potential therapeutic means to treat obesity and metabolic diseases (Cypess and Kahn, 2010; Boss and Farmer, 2012). By high-throughput screening over 3000 compounds in differentiated 3T3-L1 and C3H10T1/2 adipocytes to find small-molecule compounds capable of activating thermogenesis, Lu et al have identified artemether, an artemisinin derivative, as an activator of browning and thermogenesis *in vitro*. Further examination reveals artemisinin and other artemisinin derivatives, dihydroartemisinin, artesunate and arteether, could also promote browning. Importantly, local delivery of artemether into subcutaneous fat or systemic delivery via tail vein in mice effectively reduces high fat diet induced body weight gain, enhances cold tolerance and improves insulin sensitivity. Mechanistic study indicates that p38 MAPK/ATF2 axis and Akt/mTOR pathway are partially responsible for the browning effects of artemether (Lu et al., 2016). In another study, it is reported that leaf extracts of Artemisia annua, the source of artemisinin, attenuates hepatic steatosis and inflammation in diet-induced obese (DIO) mice (Kim et al., 2016). These studies shed first light on the possibility of exploiting artemisinin and its derivatives as an anti-obesity and anti-fatty liver drug *in vivo*, though detailed mechanistic study and clinical data are warranted. As a paradigm of the translational application of TCM, continued characterization of the metabolic properties of artemisinin and its derivatives would bring hope to patients with obesity and metabolic diseases.

### Curcumin

In sharp comparison with artemisinin, whose effectiveness is well acknowledged in academia, curcumin may be the most controversial TCM compound in the eyes of western chemists and biologists. Curcumin is the principal curcuminoid in the turmeric of the ginger family. There are thousands of reports and over 120 clinical trials studying the effectiveness of curcumin in various diseases, including erectile dysfunction, hirsutism, baldness, cancer, and Alzheimer’s disease, yet the results fail to reconcile with one another. The contradictions are probably due to the poor solubility of curcumin in aqueous solution, thus limiting its bioactivity and stability in many studies (Nelson et al., 2017). When interpreting results from various studies, one has to keep in mind that, on one hand, substantial evidences exist in a large body of literatures about curcumin’s biological activities under different circumstances and its effectiveness both *in vitro* and *in vivo* (Heger, 2017). But on the other hand, it is of major concern that curcumin fluoresces naturally, which may interfere with drug screening that relies highly on fluorescence signals, producing screen artifacts and generating false positive results (Baker, 2017). Thus, any attempt at the pharmacological use of curcumin should take these factors into account.

In the metabolic aspects, in adipocytes, consistent reports indicate that curcumin suppresses adipocyte differentiation by affecting classic regulators of adipogenesis (Ejaz et al., 2009; Kim et al., 2011; Sakuma et al., 2017). Curcumin also ameliorates hypoxia-induced insulin resistance and inflammation in 3T3-L1 adipocytes (Priyanka et al., 2017). In the browning process, it has been shown that curcumin treatment induces browning in primary white adipocytes and adipose tissues and augments thermogenic and mitochondrial gene programs in a classic norepinephrine dependent manner (Wang et al., 2015; Lone et al., 2016). In rodents, curcumin treatment enhances energy expenditure in DIO murine models and protects against weight gain and inflammation of adipose tissues (Weisberg et al., 2008; Shao et al., 2012). In a few clinic trials, curcumin has been shown to improve insulin resistance and hyperlipidemia in patients with metabolic syndromes (Mohammadi et al., 2013; Ganjali et al., 2014), while others produce negative results (Nelson et al., 2017).

Importantly, the resolution to the predicament of insolubility and instability of curcumin, which hinders its clinical application, may lie in the researches of curcumin analogs. For example, curcumin-3, 4-dichloro phenyl pyrazole (CDPP) shows significantly improved bioactivity while retains curcumin’s capability in suppressing adipocyte differentiation and preventing hyperlipidemia in DIO rodents (Gupta et al., 2017). C66, a novel curcumin derivative, inhibits JNK phosphorylation, reduces high glucose-induced inflammation in cardiomyocytes and prevents the development of diabetic cardiomyopathy in mice (Pan et al., 2014). Alternatively, nano-formulated curcumin generated using nanoparticles has shown improved bioactivity and efficacy compared to native curcumin in various *in vitro* and *in vivo* disease models, indicating its potential implication in metabolic studies (Rahimi et al., 2016). However, despite the metabolic benefits in curcumin-treated adipocytes and mice models, these studies lack detailed mechanisms and target molecules. Further mechanistic investigations are needed to disperse the controversy around curcumin before it can be further implicated in the treatment of obesity and metabolic diseases.

## Multi-Targets Vs. Single Target: Celastrol and Capsaicin

Mechanistic studies are vital in drug development to prevent potential side effects. Compounds with single targets are favored by scientists for their balanced efficacy and safeness, although compounds with multiple targets in multiple organs could be of potential interest in treating obesity and metabolic diseases since the patients usually feature pathological changes in not one but multiple organs, i.e., adipose tissues, liver and muscle. As an example, celastrol is a TCM compound targeting multiple metabolic organs with detailed mechanisms of action deciphered (Liu et al., 2015; Ma et al., 2015; Hu et al., 2017). In comparison, capsaicin is well known for functioning through its receptor TRPV1 (Caterina et al., 1997), the mechanism of which has been extensively studied.

### Celastrol

Celastrol (tripterine) is a chemical compound isolated from the root extracts of Tripterygium wilfordii (Thunder god vine, TGV). As a TCM, TGV has wide and longtime application in treating inflammatory diseases in patients, i.e., rheumatoid arthritis. As one of the major active ingredients of TGV, celastrol has multiple functions in anti-oxidation, anti-inflammation, anti-neurodegeneration, and anti-cancer, though its anti-inflammation effects are most extensively studied (Cascão et al., 2017). Recently, a series of studies have brought celastrol onto the metabolic stage and highlight it as a versatile regulator of obesity and metabolic diseases with both central and peripheral targets. Centrally, Liu et al. (2015) has reported that celastrol functions as a leptin sensitizer to reduce food intake in DIO mice and possibly treats obesity by both activating leptin receptor-STAT3 pathway and inhibiting NF-κB in the hypothalamus. Peripherally, Zhang’s group has demonstrated that under inflammatory conditions, celastrol binds to Nur77, an orphan nuclear receptor and an inducer of mitochondria apoptosis, and promotes its translocation into mitochondrial for inflamed mitochondrial autophagy and clearance, thus reduces inflammation and improves hepatic steatosis in mice (Hu et al., 2017). Celastrol could also induce Sirt1 transcription and alleviate hepatic steatosis by decreasing SREBP1 and increasing AMPKα expression (Zhang et al., 2016). Another report from Luo et al. (2017) reveals that in liver and adipose tissues of DIO mice, celastrol suppresses inflammation by reducing macrophage M1 polarization via its regulation on the Nrf2/HO-1, MAPK, and NF-κB pathways.

Apart from its impact on obesity and metabolic diseases through inflammatory inhibition in multiple organs, celastrol also exerts its beneficial effects by directly promoting energy expenditure. HSF1 is a classic transcription factor induced by multiple stimuli, including heat shock, oxidative and mechanical stresses (Anckar and Sistonen, 2011). It orchestrates the function of heat shock proteins in protein refolding and damage repair, thus is vital for protein homeostasis and cell survival (Gomez-Pastor et al., 2018). Interestingly, HSF1 could promote mitochondrial biogenesis and adaptive thermogenesis via its interaction with and transactivation of PGC1α, the master regulator of energy metabolism that was previously thought to be mainly induced by cold exposure (Ma et al., 2015). Celastrol activates HSF1 as shown by HSF1 phosphorylation and activation of its downstream target genes (Westerheide et al., 2004). Based on these preliminary data and the fact that celastrol is derived from TCM with wide application in humans, Ma et al. (2015) test its metabolic effects and reveal that celastrol prevents weight gain, improves hepatic steatosis and ameliorates insulin resistance in mice fed a high fat diet. Intriguingly, in this study, celastrol blocks obesity progression in mice without reducing food intake or affecting hypothalamus metabolic genes expression, possibly because these mice didn’t develop severe leptin resistance as the DIO model used in Liu’s paper. Instead, celastrol activates the HSF1-PGC1α axis in peripheral organs to induce browning of white fat and promote mitochondrial function in muscle. Celastrol treatment loses these beneficial effects in HSF1 and PGC1α deficient cells and mice, suggesting that HSF1-PGC1α axis is at least partially responsible for the metabolic function of celastrol in the peripheral organs (Ma et al., 2015).

In patients with obesity and metabolic diseases, multiple organs, i.e., adipose tissues, liver, muscle and brain experience pathological changes. It has been shown that celastrol targets multiple organs and improves their metabolic performances simultaneously, thus rendering it a TCM compound with comprehensive effects in treating obesity and metabolic diseases (Ma et al., 2015). Future work could be focused on its structural modification to obtain celastrol derivatives that preferentially target specific organs. Besides, it is of note that triptolide, another major active component of Tripterygium wilfordii, shows similar anti-inflammatory and anti-cancer effects to celastrol (Li et al., 2014). Combined treatment of triptolide and celastrol shows synergistic effects in suppressing tumor cell growth *in vitro* (Jiang et al., 2015). It would be worthwhile to test their synergistic effects in the treatment of obesity and metabolic diseases. Finally, although celastrol treatment of various concentrations and lengths of time shows no overt side effects or toxicity in rodents, low sperm density and some toxicity are reported in male patients treated with TGV for rheumatoid arthritis (Lopez et al., 2005). Future studies are warranted to elucidate the efficacy and a safe concentration of action for celastrol and other TGV constituents in clinic.

### Capsaicin

One hundred years ago, Capsaicin was identified and purified as one of the major active biological components of peppers (Capsicum). Peppers taste spicy by inducing the “hot” sensation to different extents and are widely used in daily life as a seasoning. They also have longtime use in TCM to relieve physical pain and treat gastrosis, detrusor hyperreflexia and rheumatic arthritis. Among active TCM compounds translated for pharmaceutical use, capsaicin is one of the best examples featuring extensively studied molecular targets and mechanisms. Unlike celastrol, which exerts its function through multiple targets in diverse organs, capsaicin functions through a single and clear target, its receptor TRPV1 (Caterina et al., 1997). TRPV1, a Ca^2+^ ion channel highly enriched in a specific subset of peptidergic sensory neuron in human and rodents, was identified and cloned in 1997 (Gunthorpe and Szallasi, 2008). It is a prime target for pain relief upon activation by capsaicin, as well as thermal heat, acidic conditions and allyl isothiocyanate (Gavva, 2008). TRPV1 antagonists reduce pain by blocking receptor signal transduction, but their clinical application is hurdled by the simultaneous induction of hyperthermia by TRPV1 blockade, potentially due to TRPV1’s function in maintaining body temperature in central nervous system (Cui et al., 2006). It is interesting that long term or high dose treatment of TRPV1 agonists, such as capsaicin, cause receptor desensitization or kill the TRPV1 neurons, thus mimicking TRPV1 deficiency and leads to pain alleviation, suppression of TRPV1 mediated inflammation but without the conundrum of TRPV1 antagonists (Knotkova et al., 2008).

With the accumulation of evidence about the regulatory function of TRPV1 in the central nervous system for food intake and body temperature, as well as in peripheral organs for insulin and adipokine secretion, agonist-mediated TRPV1 desensitization begins to attract attention for its potential application in metabolic field. Capsaicin, as the classical TRPV1 agonist, has been shown in human and in rodents to increase satiety, reduce food intake, increase sympathetic nervous system activity (Reinbach et al., 2009; Janssens et al., 2014), and not surprisingly, enhance lipid metabolism (Kang et al., 2010) and activate thermogenesis (Baskaran et al., 2017). Mechanically, these reports consistently demonstrate that the metabolic effects of capsaicin no longer exist when TRPV1 is deficient, though capsaicin may also induce the Sirt1/PRDM16/PPARγ axis for its browning effects on white fat (Reinbach et al., 2009; Kang et al., 2010; Janssens et al., 2014; Baskaran et al., 2017). These findings signify the important potential of capsaicin and TRPV1 in the metabolic aspect. It has to be noted that other TPRV family members, i.e., TRPA1, TPRM8, and TRPV4, could be activated by different agonists/antagonists or sense different stimuli to exert various metabolic regulation, a particular intriguing example being TRPV4, which functions as a negative regulator of thermogenesis (Ye et al., 2012). Thus, aside from capsaicin, TCM would serve as a valuable source to screen more active compounds that target different TRPVs to open new therapeutic avenues in treating obesity and metabolic diseases.

## Common Vs. Rare: Berberine and Ginsenosides

Traditional Chinese medicine contains medical extracts from a wide range of plants, animals and mineral products. In the long list, some are common everyday plants while some are rare and hard to cultivate. In drug development, after primary considerations like efficacy and safety are met, low development cost would be an advantage. A good example is Coptis chinensis and its active ingredient berberine, a common OTC drug with high popularity vs. ginseng, a TCM shrouded in mysterious atmosphere because of its effectiveness and rarity, though its development as a modern drug is hindered by its complex ingredients, ginsenosides.

### Berberine

Coptis chinensis, a common and easy to cultivate medicinal herb, is one of the most widely used TCM with versatile effects since ancient China. The famous ancient Chinese proverb “a bitter medicine cures the disease,” the bitter medicine referring to Coptis chinensis, exemplifies its long history of use and popularity in people. Coptis chinensis is routinely prescribed to treat bacteria induced diarrhea for its antibiotic properties. Today, it has been identified that the major active ingredient in Coptis chinensis is berberine. With the development and refinement of advanced synthesis technology, berberine is produced in large quantities and at low cost, thus becoming a standard collection in the medicine cabinet of most Chinese families. Besides diarrhea, *in vitro* and *in vivo* studies suggest that berberine is a potential drug in the treatment of type 2 diabetes mellitus, hyperlipidemia, and certain types of cancer (Liu et al., 2016). As early as 2004, Jiang’s group has found that berberine binds to the 3′UTR of LDLR mRNA, resulting in increased LDLR stability, enhanced hepatic LDL assimilation and reduced cholesterol level. In hypercholesterolemic patients and hyperlipidemic hamsters, berberine treatment significantly lowers total cholesterol, LDL and triglyceride levels with a mechanism of action distinct from Statins, a classic cholesterol-lowering drug targeting HMG-CoA reductase (Kong et al., 2004). Later, Ning’s group showed the effectiveness of berberine in lipid and glycemic control in larger cohort of patients with Type 2 diabetes and hyperlipidemia based on comprehensive metabolomics (Zhang et al., 2008; Gu et al., 2010). Furthermore, berberine is shown to activate thermogenesis in adipocytes through the AMPK-PGC1α axis, which leads to increased energy expenditure, reduced weight gain and improved cold tolerance in obese db/db mice (Zhang et al., 2014).

Of note, berberine also exerts anti-aging function by mechanisms inhibiting mTOR/S6 pathway via AMPK activation, as well as reducing the endogenous ROS level and constitutive oxidative DNA damages through NRF2 (Halicka et al., 2012). In Drosophila melanogaster, berberine prolongs life span and stimulates locomotor activity potentially by blocking kynurenine formation from tryptophan, which is associated with aging (Navrotskaya et al., 2012). Since it has been shown that metabolic improvements are one of the major drives for longevity (Canaan et al., 2014; Ma et al., 2014), it would be interesting to assess how much of the extended longevity is contributed by berberine’s promotion of metabolic health in the future and whether mammalian lifespan is also affected by berberine treatment.

In marked contrast to its effective in some clinical trials, the plasma level of berberine is found to be fairly low in patients (Hua et al., 2007), indicating that besides the classic pharmacological model, other factors might also in play in exerting berberine’s beneficial effects. Promoted by sophisticated sequencing technologies, there has been a rapid growth in gut microbiota researches, spotlighting its critical involvement in the progression of multiple diseases including obesity and metabolic diseases. Due to its antimicrobial activity and poor solubility and absorption in the gut, berberine is put under the spotlight for its impacts on gut microbiota. As demonstrated by several independent groups, berberine improves high fat diet induced obesity and metabolic diseases through intestinal microbiota modulations (Han et al., 2011). Berberine treatment causes a structural change in gut microbial flora, largely reduces its diversity by enriching a few short-chain fatty acid (SCFA) producing bacteria including Blautia and Allobaculum, in turn elevates SCFA levels in the intestine and alleviates host inflammation (Zhang et al., 2012). Berberine also affects bile acid metabolism via its modulation on intestinal flora, by inhibiting cholic acid 7α-dehydroxylation conversion into deoxycholic acid (Gu et al., 2015). Interestingly, in ApoE^-/-^ atherosclerosis mice model, in addition to inducing a higher Akkermansia abundance in the gut flora, berberine administration is also found to preserve gut barrier integrity by increasing intestinal epithelial tight junction and colon mucus layer thickness in high fat diet-fed mice (Zhu et al., 2018). Last but not least, berberine could potentially promote metabolic health by playing a role in the microbiota-gut-brain axis. For example, berberine increases serum Glucagon-like Peptide-1 (GLP-1) and Neuropeptide Y level while decreases Orexin A level, which are all gut-brain peptides critical in satiety and energy homeostasis (Sun et al., 2016). Of note, GLP1 receptor is found to be elevated in the hypothalamus of berberine-treated mice, suggesting central nervous system might be another target for metabolic regulation by berberine (Sun et al., 2016).

### Ginsenosides

Hidden in deep mountains, and difficult to find, ginseng is regarded by Chinese people as a rare and precious diet supplement to strengthen holistic health. Modern studies have shown that ginseng has anti-oxidant and anti-inflammation properties in cardiovascular and central nervous system, thus favoring healthy aging. The active ingredients in ginseng are ginsenosides, a class of steroid glycosides and triterpene saponins. There are more than 30 biologically active ginsenosides in ginseng, i.e., protopanaxadiols and protopanaxatriols, that all retain the beneficial effects of ginseng to some extent, thus it would be interesting to systemically assess the similarities and differences of these ginsenosides.

Numerous studies have demonstrated that ginsenosides are effective in preventing obesity, hyperlipidemia, hyperglycemia, and hepatic steatosis in DIO mice and rats, their action of targets including adipose tissue, liver, muscle and brain. In 3T3-L1 adipocytes, ginsenosides Rb1, Rg3, Rh1, Rf, and Re etc. suppress adipocyte differentiation by inhibiting the classical adipogenic transcription regulators PPARγ and C/EBPs (Lee et al., 2011; Gu et al., 2013; Siraj et al., 2015; Koh et al., 2017). In one independent research, Rb1 is shown to induce browning effects in 3T3-L1 adipocytes by increasing PPARγ activity, which is abrogated by PPARγ antagonist GW9692 treatment (Mu et al., 2015). Ginsenosides Rb1, Rg1, Rg5, and Re also target skeleton muscle for enhanced insulin sensitivity and cardiomyocytes for improved cardiac functions (Xie et al., 2006; Guan et al., 2017; Peng et al., 2017; Xiao et al., 2017b). In liver, Rg1, Rg3, Rg5, and Rb2 prevent hepatic steatosis with AMPK as their possible common target (Shen et al., 2013; Huang et al., 2017; Lee et al., 2017; Xiao et al., 2017a). There are also a few researches report that ginsenosides may target central nervous system in obese mice to improve leptin sensitivity in cortex and relieve central inflammation in hypothalamus (Wu et al., 2014).

Despite their consistent effectiveness in rodents and cellular models, meta-analysis of recent clinical trials assessing ginseng effects in cardiovascular diseases and metabolic diseases reveal that around 65% of the studies show significant improvements in ginseng treated groups, while the rest produce negative results (Kim et al., 2015). For instance, one study has shown that ginseng itself and ginsenoside Re fail to improve β-cell function or insulin sensitivity in overweight and obese population with diabetes (Reeds et al., 2011). These inconsistencies might be due to the vast variants of ginseng species in North America and East Asia and the complexity of biologically active ginsenosides in different ginsengs. It would be worthwhile to invest more energy in isolating and characterizing the function of individual ginsenoside from various ginseng species, based on which more accurate clinical trials could be performed to assess their therapeutic potential in patients. Plus, developing efficient synthetic routes to obtain specific ginsenoside of interest would be of importance since extracting ginsenosides from ginseng might be expensive and low yield.

### Perspectives

**FIGURE 1:**
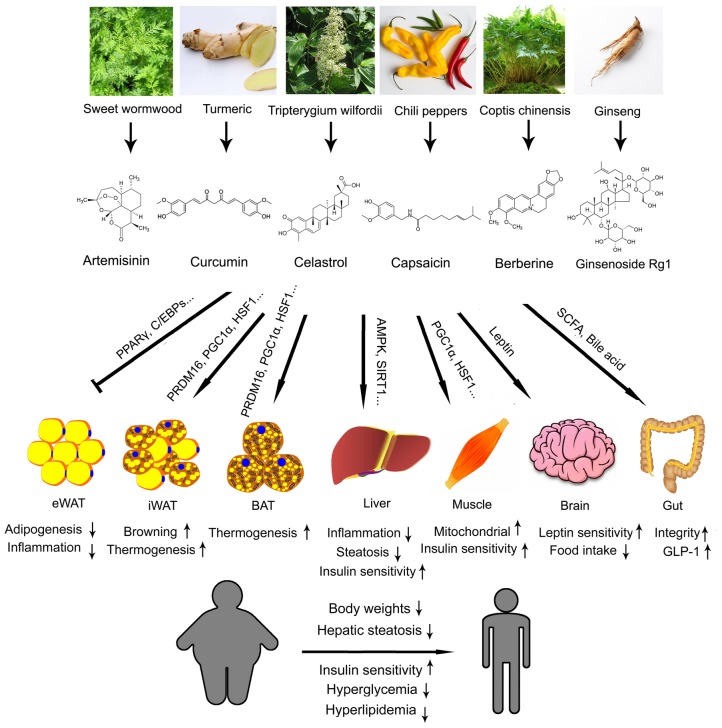
Illustration summary of the original Traditional Chinese medicine (TCM) herbs, coumpound structures, metabolic effects and major mechanisms of artemisinin, curcumin, celastrol, capsaicin, berberine, and ginsenoside. eWAT, epidydimal white adipose tissue; iWAT, inguinal white adipose tissue; BAT, brown adipose tissue.

In this review, we have highlighted six compounds derived from TCM, artemisinin, curcumin, celastrol, capsaicin, berberine and ginsenoside, and evaluated various aspects of their property and their potentials in treating obesity and metabolic diseases (**Figure 1**). There are substantial clinical data on the safeness and side effects of artemisinin during its implication as a standard treatment for malaria. This makes it relatively easy to be transformed into a metabolic drug, although more data have to be obtained on its impact on metabolic parameters. On the other hand, clinical trials using curcumin have produced controversial results, which urge for more randomized, double-blind, parallel controlled, multi-center clinical trials for fair judgment before it could be put into use. Celastrol, capsaicin and berberine are all promising novel therapeutics against obesity and metabolic diseases for their convincing effectiveness on metabolic improvement from *in vitro* and *in vivo* studies. Clinical data is required to access their efficacy and side effects on patients in the future. Ginseng, though famous for its holistic effects, has to be carefully analyzed to identify the detailed functions of individual ginsenosides in metabolism. Then, efforts are needed to find a cheaper way to synthesize the desired active ginsenosides. Besides the six compounds reviewed here, more TCM ingredients are waiting to be re-discovered and developed as novel drugs targeting obesity and metabolic diseases.

Along the history of mankind’s everlasting pursuit for health, TCM plays an indispensable role with its unique yet empirical theory in diagnosing and treating diseases, which still holds significance in the modern point of view. Like the increased acceptance and popularity of Chinese ancient philosophy in western world, TCM theory is attracting more and more attention from the western medical establishment. For instance, TCM acknowledges the crosstalk among different organs and considers human body as a whole. Rather than targeting the affected parts alone, TCM strives to fight diseases in an integrated way. Its focus on complex interactions within biological systems coincides with the core idea of “Systems Biology,” which is a powerful and fundamental tool for researches on subjects as complicated as human bodies. Secondly, similar to “Precision Medicine,” TCM treatment is highly personalized. Ailments as simple as cold or headache are characterized based on different pathogenesis and handled accordingly, rather than using a standard symptom-based protocol as commonly practiced in western medicine. Other factors like patients’ gender, sex, age, living condition, and life style all play a role when design a prescription in TCM. It may seem chaotic at first impression, but it is clear now that a disease, especially obesity and metabolic diseases, is a highly interactive outcome of one’s genetic and environment, thus the diagnostic strategy of TCM is not without solid foundation, although detailed mechanisms behind TCM theory have to be clearly addressed before it could be put into modern use. Thirdly, TCM formula is mostly Fufang (empirically combining multiple medicinal extracts to achieve best efficacy against a disease), which is similar to “Combination Therapy” in modern medicine. Like in the case of celastrol and triptolide, TCM Fufang formula is a rich source to discover synergistic effects of numerous active ingredients to develop new combination therapy paradigm of better curative effects. Of course, similar to western drugs, TCM compounds have to be carefully reviewed and tested to decide the ideal treating strategy and eliminate side effects before they could be used as a therapeutic. To this end, a solid research and development protocol is indispensable to unravel the ancient secrets hidden in TCM to benefit patients in the modern world, at the same time prevents sporadic yet notorious incidence like *Aristolochia* and aristolochic acids in liver cancer (Ng et al., 2017).

Regarding the six candidate compounds discussed in this review, although *in vitro* mechanistic studies are extensive, it is of note that the effects of artemisinin and celastrol on metabolic parameters are evaluated in cellular and rodent models, which have limited extrapolation in human and require further clinical tests. Clinical studies on capcaisin and berberine produced positive data on metabolic fitness yet more studies are warranted, whereas the inconsistencies within the numerous clinical trials on curcumin and ginsenosides need reconciling. It is important to focus future studies on addressing these points to promote their translation into pharmaceuticals against obesity and metabolic diseases.

In summary, though great efforts are still needed to better understand its mechanisms and clinical relevance, TCM possesses great potential as a vast and readily available source for finding and developing new drugs against obesity and metabolic diseases. With the high prevalence of obesity and metabolic diseases in population and the resulting high cost for diseases care, it would be wise to devote more resources in researching of TCM for new therapeutic inspirations.

## Author Contributions

LX and XM conceived the review and LX, WZ, DW, and XM wrote the manuscript.

## Conflict of Interest Statement

The authors declare that the research was conducted in the absence of any commercial or financial relationships that could be construed as a potential conflict of interest.

## Abbreviations

AMPK: Protein kinase AMP-activated catalytic subunit alpha 1
ATF2: Activating transcription factor 2
C/EBP: CCAAT/enhancer binding protein
HO-1: Heme oxygenase-1
HSF1: Heat shock factor 1
LDLR: Low density lipoprotein receptor
MAPK: Mitogen activated kinase-like protein
NRF2: Muclear factor erythroid 2
PGC1α: Peroxisome proliferative activated receptor gamma coactivator 1 alpha
PPARγ: Peroxisome proliferator activated receptor gamma
PRDM16: PR/SET domain 16
Sirt1: Sirtuin 1
SREBP1: Sterol regulatory element binding protein 1
STAT3: Signal transducer and activator of transcription 3
TPRM8: Transient receptor potential cation channel subfamily M member 8
TRPA1: Transient receptor potential cation channel subfamily A member 1
TRPV1: Transient receptor potential cation channel subfamily V member 1
TRPV4: Transient receptor potential cation channel subfamily V member 4
UCP1: Uncoupling protein 1
